# Muscle Synergies Reliability in the Power Clean Exercise

**DOI:** 10.3390/jfmk5040075

**Published:** 2020-10-22

**Authors:** Paulo D. G. Santos, João R. Vaz, Paulo F. Correia, Maria J. Valamatos, António P. Veloso, Pedro Pezarat-Correia

**Affiliations:** 1 Neuromuscular Research Lab, Faculty of Human Kinetics, Lisbon University, 1495-751 Cruz Quebrada, Portugal; pauloduarteguiasantos@gmail.com (P.D.G.S.); paulo.correia.performance@gmail.com (P.F.C.); mjvalamatos@fmh.ulisboa.pt (M.J.V.); ppezarat@fmh.ulisboa.pt (P.P.-C.); 2 CIPER, Faculty of Human Kinetics, Lisbon University, 1495-751 Cruz Quebrada, Portugal; apveloso@fmh.ulisboa.pt; 3 Biomechanics and Functional Morphology Laboratory, Faculty of Human Kinetics, Lisbon University, 1495-751 Cruz Quebrada, Portugal

**Keywords:** strength training, neural adaptations, muscle coordination, reliability, electromyography

## Abstract

Muscle synergy extraction has been utilized to investigate muscle coordination in human movement, namely in sports. The reliability of the method has been proposed, although it has not been assessed previously during a complex sportive task. Therefore, the aim of the study was to evaluate intra- and inter-day reliability of a strength training complex task, the power clean, assessing participants’ variability in the task across sets and days. Twelve unexperienced participants performed four sets of power cleans in two test days after strength tests, and muscle synergies were extracted from electromyography (EMG) data of 16 muscles. Three muscle synergies accounted for almost 90% of variance accounted for (VAF) across sets and days. Intra-day VAF, muscle synergy vectors, synergy activation coefficients and individual EMG profiles showed high similarity values. Inter-day muscle synergy vectors had moderate similarity, while the variables regarding temporal activation were still strongly related. The present findings revealed that the muscle synergies extracted during the power clean remained stable across sets and days in unexperienced participants. Thus, the mathematical procedure for the extraction of muscle synergies through nonnegative matrix factorization (NMF) may be considered a reliable method to study muscle coordination adaptations from muscle strength programs.

## 1. Introduction

When performing a motor task, the Central Nervous System (CNS) has to control the biomechanical redundancy established by infinite neuromuscular interactions, in a way that all muscles involved can lead to the desired joint moments and assure that the task is successfully performed [1]. The complexity of the CNS to control all the involved elements is not completely understood yet. It has been suggested that there might be a mechanism that deals with the many degrees of freedom available in the neuromusculoskeletal system. Such mechanism would consist in the presence of low-dimensional elements, muscle synergies, that decrease the computational burden and, hence, would allow a more efficient control from the CNS [2]. The muscle synergies allow the CNS to control smaller number of variables, simplifying the construction of motor behaviors [3,4,5]. Recent research suggest that muscle synergies represent motor modules encoded in spinal cord and brainstem, and controlled by motor cortical areas and integrating sensory information that activate groups of muscles to generate a specific motor output [6,7]. These are considered coordinative primitives that have a neural origin and are structured in the brainstem or spinal cord [6]. Although there is growing evidence that these modules have a neural origin [8,9,10,11,12], some authors argue that these are a result of biomechanical constraints [13].

Regardless of its origin, muscle synergies may ultimately reflect muscle coordination strategies [14]. The study of muscle synergies, extracted from surface electromyographic signals (EMG), has shown to be relevant in neurorehabilitation offering the clinician a better view of the neural structure of motor behaviors and being a metric that allows to discriminate pathological changes in the nervous system [4]; and in robotics by providing the application of the muscle synergy hypothesis to develop artificial limbs [15,16]. In sports field, the low-level control of complex movements has provided useful information to improve athletes’ performance and training. The extraction of muscle synergies may provide information about how the CNS recruits the muscles during motor tasks, by reducing the dimensionality of muscle control [16]. This approach has previously been used to study human movement in crawling [17], walking and running [18], postural control [19], pedaling [20,21], rowing [22], gymnastics [23], swimming [24], and bench press [25].

A necessary next step in this field is the deeper understanding of the effect of training in these coordination strategies assessed through muscle synergy analysis. For example, a recent study showed that 4 weeks of lower limb proprioceptive training altered the modular organization of the synergies, comprising lower limb muscles, in the early phase of a single-leg drop-landing task [26]. The authors propose that this change was caused by the emergence of a new synergy composed by the plantarflexors and ankle evertors muscles. This indicates that training may induce changes in modular organization of muscle recruitment, altering, in this case, the specific spatiotemporal recruitment of plantarflexors and ankle evertors. In another study, Kristiansen and colleagues [25] extracted muscle synergies from muscles of lower-limb, upper-limb and trunk. They showed that experienced powerlifters exhibit larger inter-subject variability in the muscle vectors (i.e., individual contribution of each muscle to each synergy) compared to untrained individuals concerning the bench press exercise. Interestingly, a follow-up study from this group revealed that after a 5-week training protocol, the training group exhibited a larger inter-subject variability compared to baseline regarding the observed decreases in intra-group correlation-values [27]. These findings are not very surprising because the mechanical degrees of freedom in a bench press task is small. Therefore, a crucial step in the study of the effect of strength training in muscle synergies requires a whole-body task that involves more joints from both the upper and lower limb muscles.

The power clean, for instance, is one of the most utilized exercises in strength and power training in sports. This exercise involves high angular velocities in upper- and lower-limb joints, while the trunk stabilization is also a key point. Thereby, this exercise requires more accurate motor control of broader degrees of freedom compared to bench press, which involves movement in two joints only. Thus, before conducting an intervention study to explore the effect of strength training in this complex task, a reliability study is required to better and properly interpret possible results from a training protocol. Therefore, the aim of the present study was to investigate the intra- and inter-day reliability of the muscle synergies and the individual EMG signals during the power clean exercise in unexperienced individuals. Based on previous research about the reliability of muscle synergies [28,29] and considering the high robustness of the number of synergies observed in sports activities across different levels of expertise and conditions [21,22,23,24,25], we hypothesized that the number of muscle synergies would remain the same, while muscle synergies components and individual EMG would exhibit moderate-to-high values of intra- and inter-day reliability.

## 2. Materials and Methods

### 2.1. Participants

Twelve male participants (age 24.5 ± 2.3 years, height 1.71 ± 4.4 m, body mass 68.2 ± 6.5 kg) with five repetition maximum (5 RM) in power clean of 53.3 ± 9.8 kg (first session) and 53.2 ± 11.5 kg (second session) participated in this study. The inclusion criterion was set to select only healthy participants without prior knowledge about the critical points of the exercise. The participants were informed to abstain from any physical activity during the day before the evaluation sessions. All the participants provided informed written consent. This study was approved by the Ethics Committee of the Faculty of Human Kinetics, Lisbon (CEFMH 4/2018, permission code, 29 March 2018) and all procedures adhered to the Declaration of Helsinki.

### 2.2. Experimental Approach

The participants were assessed while performing the power clean. This exercise is commonly used for training and assessment of physical capacities and it has been previously shown to be a reliable indicator of performance in unexperienced [30] subjects. Each participant performed three sessions, with the exception of one participant that did not perform the last one. In the first session, the participants were familiarized with the task, technical learning of the movement and laboratory environment. The subsequent session was conducted approximately one week after the familiarization. The third and last session took place three to seven days after the session 2. The tests performed during sessions 2 and 3 were used to investigate the intra- and inter-session reliability.

### 2.3. Data Collection and Materials

After the warm-up, the participants were tested in power clean 5 RM. The lifted load in each set was increased by 2.5–5 kg until the 5 RM load was determined. The participants had four minutes rest between sets, and after the 5 RM was found, they had approximately one hour to recover while we placed the EMG electrodes.

An eight-camera system (Qualisys, Gothenburg, Sweden) was used and placed around the laboratory where the exercise was performed. Three markers were attached to each side of the barbell to measure its displacement. Movement data were sampled at 200 samples/s. The data collection of myoelectrical signals was recorded on sixteen muscles of the right side of the body: upper trapezius (TS), pectoralis major (PM), biceps brachii (BB), triceps brachii lateral head (TB), flexor digitorum superficialis (FDS), extensor digitorum communis (EDC), latissimus dorsi (LD), erector spinae (ES), rectus abdominis (RA), external oblique (OE), gluteus maximus (Gmax), vastus lateralis of quadriceps (VL), biceps femoris long head (BF), semitendinosus (ST), lateral gastrocnemius (GL) and tibialis anterior (TA). The electrodes were placed according to SENIAM (Surface EMG for Non-Invasive Assessment of Muscles) recommendations [31], with exception of FDS, EDC, LD, PM, RA and OE: FDS and EDC were placed as recommended by Zipp [32]; LD was positioned according to de Sèze and Cazalets [33]; PM was placed medially to the anterior axillary border; RA and OE were located 3 and 15 cm laterally from the umbilicus, respectively. Before the electrodes’ placement, the skin was shaved and cleaned with alcohol to minimize impedance. Surface EMG was acquired using sixteen bipolar surface electrodes (EMG Delsys, TrignoTM) aligned with the muscle fibers. EMG signals were preamplified and band-pass filtered between 20 and 450 Hz, while digitized at 1000 samples/s.

Participants performed one set of three repetitions with 90% of the 5 RM. Since it was not our aim to analyze the degree of muscle activation [14], we used this set for task-specific submaximal dynamic normalization of EMG signals [25]. Then, we instructed the participants to perform four sets of eight repetitions with 70% of the 5 RM. In these sets, they were asked to touch-and-go on the floor between repetitions, by performing the ascendant phase the fastest as possible and the descendant phase in a controlled manner. The start of the ascendant phase was defined in the lowest position of the barbell and the end in the highest position of the barbell. The start and end of the descendant phase were defined in the opposite way. Each phase was time normalized to 100% to account for individual differences.

### 2.4. Data Processing

The barbell displacement’s signal was smoothed with a low-pass filter (8 Hz, 4th order Butterworth). The first and last repetitions of all sets were excluded. Raw EMG signals were band-pass filtered (20–450 Hz), rectified, smoothed with a low-pass filter (12 Hz, 4th order Butterworth) and normalized to the average value of the 100 ms across the EMG peak of the set of 3 repetitions with 90% of the 5 RM. The linear envelopes of each phase were interpolated to 100 points.

### 2.5. Extraction of Muscle Synergies

Extraction of muscle synergies has been performed through NMF, implementing the algorithm proposed by Lee and Seung [34]. NMF minimizes the residual Frobenius norm between the initial matrix and its decomposition, given as in the Equations (1) and (2):

(1) E=WC+e

(2) $$\underset{\begin{array}{c} W\geq 0\\ C\geq 0 \end{array}}{min}\;{\left|\left|E-WC\right|\right|}_{FRO}$$

where *E* is a *p*-by-*n* matrix (*p* = number of muscles; *n* = number of time points), *W* is a *p*-by-*s* (*s* = number of synergies), *C* is an *s*-by-*n* matrix and *e* is a *p*-by-*n* matrix. ${\left\|\cdot \right\|}_{FRO}$ establishes the Frobenius norm and *e* is the residual error matrix. Therefore, the two multiplication matrices in which the initial matrix is decomposed represent two components: the muscle synergy vectors (*W*), regarding the relative weighting of each muscle within each synergy, and the synergy activation coefficient (*C*), regarding the relative activation time of the muscle synergies across the power clean. The algorithm was iterated 100 times.

Each set consisted of 4 to 7 repetitions. Thus, *E* was a 13 to 16 rows by 800 to 1400 columns matrix. The analysis was iterated by varying the number of synergies between 1 and 16. The number of muscle synergies selected was dependent on variance accounted for (VAF). Therefore, the number of muscle synergies was the smaller number that defined 90% of VAF if each synergy represented at least 5% of VAF [35]. Moreover, VAF for each muscle (VAF_muscle_) was calculated, guaranteeing that the extracted muscle synergies accounted their activity pattern. A VAF_muscle_ higher than 75% was considered satisfying [23].

### 2.6. Statistical Analysis

To assess the intra-day reliability of muscle synergies, we used a two-way mixed-effects intraclass correlation (ICC (3,4)), with 95% confidence interval (CI), measuring the relative reliability of VAF and VAF_muscle_. Values of ICC were categorized as follows: 0.9–1.00, excellent; 0.75–0.9, high; 0.5–0.75, moderate; <0.5, poor [36]. Standard error of measurement (SEM) was calculated to measure absolute reliability of VAF and VAF_muscle_ [37].

To compare the similarity of the muscle synergy vectors across the four sets, Pearson’s correlation coefficient (*r*) were calculated. Thus, the *r* of each muscle synergy vector represents the average of the correlation coefficients between each pair of sets (i.e., six pairs). The similarity of the synergy activation coefficients and individual EMG patterns were assessed through the maximum cross-correlation function (*rmax*). The *rmax* can be seen as indicator of the waveform similarity, and the lag time. These two parameters were obtained using the Matlab 2015a (Mathworks Inc., Natick, MA, USA) *xcorr* function for centered data (option “*coeff*”). The lag time is determined at the maximum cross correlation function and enables the assessment of differences in timing of activation. This analysis was made by averaging the *rmax* and lag time-values of each pair of sets (i.e., for each day). *R*-values were categorized as follows: 0.7–1.0, strong correlation; 0.3–0.7, moderate correlation; <0.3, weak correlation [38].

We verified normality through the Shapiro–Wilk test. For each synergy, the differences in the average lag time of sets were evaluated performing a sample Student’s t-test with zero as reference value and the Cohen’s d as measure of effect size. If normality was not assumed, one-sample Wilcoxon signed rank test was used.

Inter-day reliability of VAF and VAF_muscle_ was assessed by using single measure ICC (3,1) for the average of the four sets of each day. ICC (3,1) was also used to measure interday reliability of the 5 RM power clean test. Regarding muscle synergy vectors and synergy activation coefficients we compared each set of the first day with each set of the second day. Then, the reliability analysis was similar to the described for intra-day analysis. We calculated the average of the sixteen values of *r* for each muscle synergy vector and the average of the sixteen values of *rmax* for each synergy activation coefficient and individual EMG patterns (*rmax* and lag times). For each synergy, the differences in the average lag time between days were evaluated performing a sample Student’s t-test with zero as reference value or one-sample Wilcoxon signed rank test when normality was not assumed.

All statistical analyses were performed in SPSS software (SPSS Inc., Chicago, IL, USA) using a significance level of *p* < 0.05.

## 3. Results

**Figure 1:**
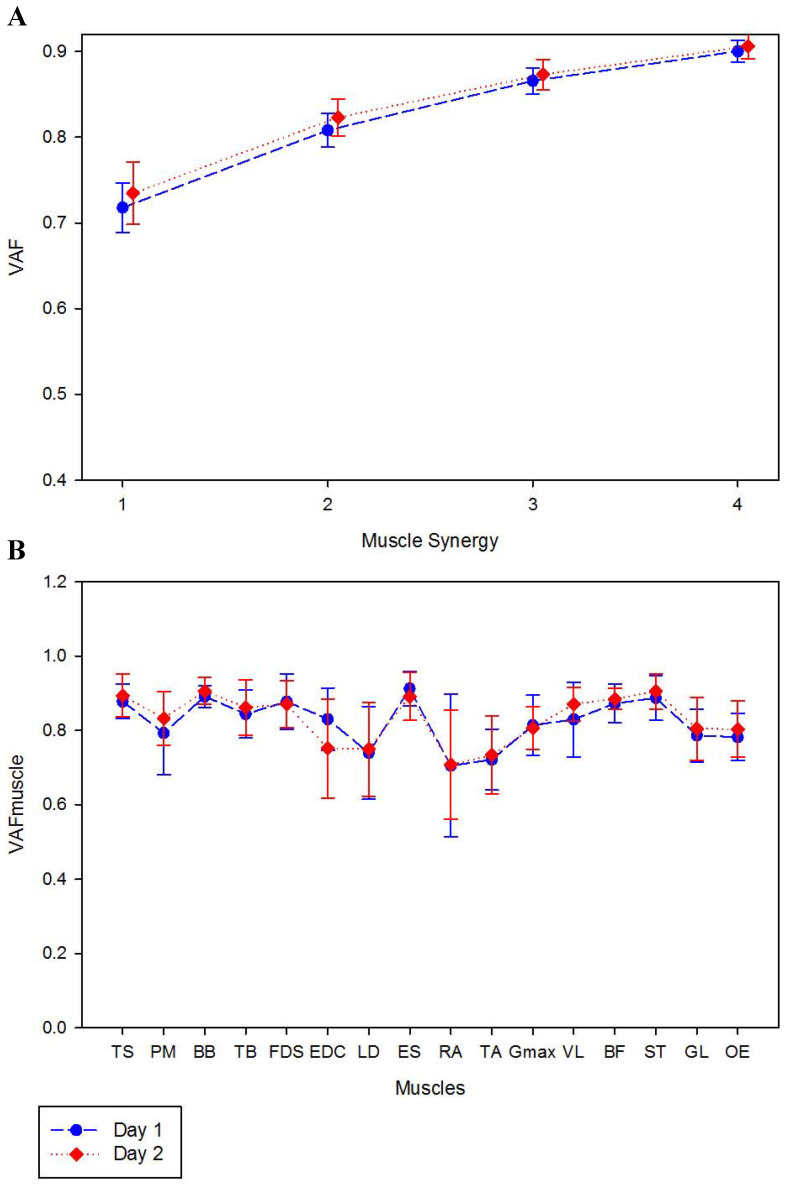
Mean values of global (**A**) and local (**B**) variance accounted for, VAF and VAFmuscle, for day 1 and 2.

Using the described criteria to identify the number of muscle synergies, all the participants exhibited three muscle synergies. The muscle synergy #1 mainly represented the back and hip extension (LD, ES, Gmax, BF, ST) and the plantarflexion (GL). The muscle synergy #2 involved the upper-limb muscles (TS, BB, TB, FDS, EDC) and the muscle synergy #3 represented the final of the ascendant phase and mainly involved the core muscles (RA, OE, ES) the PM, VL and TA. The synergy structures are depicted in Figure 1.

### 3.1. Intra-Day Reliability

The three muscle synergies represented 86.6 ± 1.6% and 87.3 ± 1.8% of VAF in first and second day, respectively (Figure 1). An excellent reliability was shown for the three extracted synergies in both days (ICC: 0.92 to 0.98) and a high to excellent reliability was shown for VAF_muscle_ (ICC: 0.80–0.98) with exception of OE in day 1 that presented only moderate values of ICC (0.65). All ICC and SEM-values are presented in Table 1.

**Figure 2:**
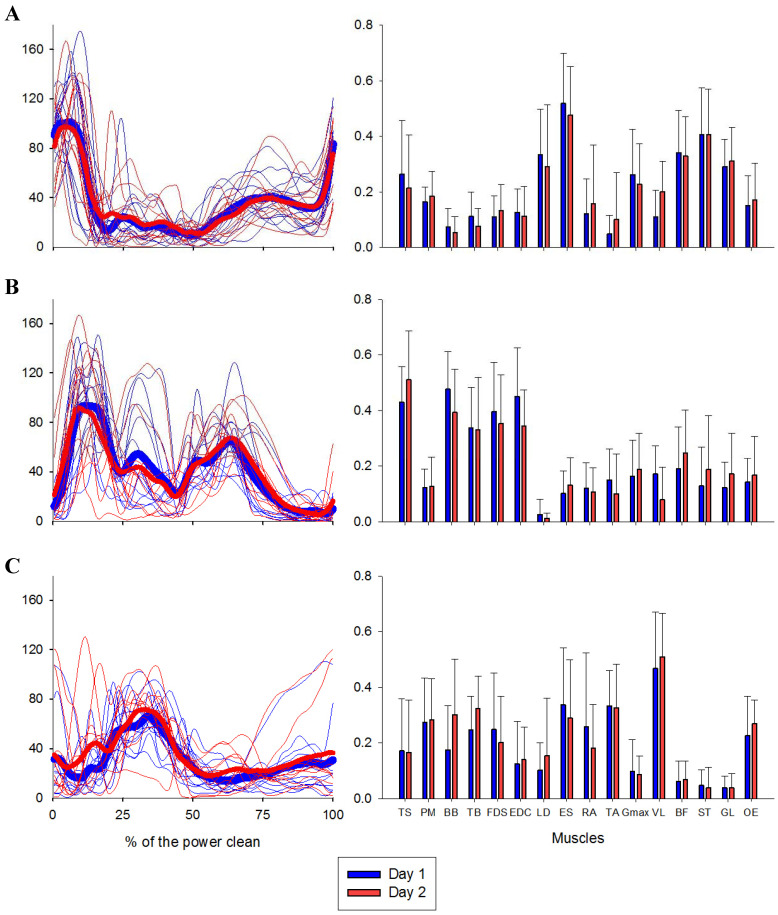
Synergy activation coefficients and muscle vectors (UA) across subjects in each day are represented in left and right panel, respectively. For the coefficients, thick lines represent the mean of each day, while the thin lines represent individual synergy activation coefficients. For the vectors, the bars represent the mean of each muscle. (**A**) regards to synergy #1, (**B**) to synergy #2 and (**C**) to synergy #3.

Regarding muscle synergy vectors on the first day, we verified strong correlations (*r*: 0.84, 0.85 and 0.74 for vector 1, 2 and 3, respectively). Moreover, on the second day, all muscle synergy vectors have shown strong correlations (0.83, 0.87 and 0.86 for vectors 1, 2 and 3, respectively). Synergy activation coefficients showed strong correlations between sets for day 1 and day 2 (0.93, 0.97). However, lag time showed to be significantly different from 0 between sets for synergy #2 in day 1. Vectors and coefficients are depicted in Figure 2, and the correlation-values are presented in Table 2 and Table 3.

The individual EMG profiles showed a very strong correlation across sets in both days, presenting *rmax*-values ranging from 0.89 to 0.99 (Table 2). However, significant lag times were observed for TS (Day 1: −0.22 ± 0.46%; Day 2: −0.33 ± 0.47%), PM (Day 1: −0.19 ± 0.33%; Day 2: −0.21 ± 0.30%), BF (Day 1: −6.87 ± 3.27; Day 2: −8.80 ± 3.48%) and ST (Day 1: −0.77 ± 0.94%; Day 2: −0.62 ± 0.71%) in both days, for GL in day 1 (−6.45 ± 8.81%) and for BB in day 2 (−0.37 ± 0.40%) (Table 2).

### 3.2. Inter-Day Reliability

The 5 RM power clean test used to determine the lifted weight by each participant showed an excellent reliability (0.97), and the SEM was 2.82 Kg.

VAF for the three synergies had moderate ICC (3,1)-values (0.66, 0,62 and 0,54 for synergy #1, #2 and #3, respectively). VAF_muscle_ exhibited poor values of ICC, excepting for Gmax and TB that had moderate (0.63) and high (0.83) reliability values, respectively.

Synergy activation coefficients showed no differences in lag time and appeared to be strongly correlated (*rmax*: 0.87, 0.90 and 0.87 for synergy #1, #2 and #3, respectively) across days. However, muscle synergy vectors presented moderate values of correlation (*r*: 0.56, 0.59 and 0.50 for synergy #1, #2 and #3, respectively). Correlation-values are presented in Table 2 and Table 3.

Individual EMG profiles presented strong correlation-values [0.89, 0.97] and did not present any lag time between days.

## 4. Discussion

The aim of this study was to evaluate intra- and inter-day reliability of muscle synergies extracted from sixteen EMG signals collected in whole-body muscles in a complex strength training task, namely the power clean exercise. We hypothesized that muscle synergies would be reliable both intra- and inter-day. Despite some minor differences in the timing of activation of some muscles in muscle synergy components, our hypothesis was globally verified. A three-synergy model was chosen, because the fourth synergy explained less than 5% of variance across sets. The total VAF-values defined for the chosen synergy model explaining less than the 90% target may be related with the complexity of motor control when performing the exercise, considering the variability between participants that showed slight differences in the variance explained by each synergy [23]. The same number of synergies was observed in other complex tasks like rowing [22], breaststroke swimming [24] or backward giant swing [23]. However, when comparing to other strength training-related research, synergy analysis showed that only two synergies explain more than 90% of total VAF in bench press exercise [25,28]. The latter authors showed that the two synergies observed were related to the concentric and eccentric phases, respectively. This difference can be explained by the number of degrees of freedom available at a certain task [23]. However, it is important to note that this comparison should be carefully made due to the chosen muscles and its number [39], and to the potential effect of different filtering techniques prior to the extraction of muscle synergies [40].

### 4.1. Intra-Day Reliability

The intra-day reliability of total VAF was excellent for the three extracted muscle synergies in both days, revealing that the participants utilized the same synergistic organization across sets. VAF_muscle_ ICC-values were high to excellent between sets, with exception of OE in first day, meaning that a three synergy-model only moderately explained the variance of the muscle. This may be related with the complex anatomy of the abdominal region and adipose tissue around the abdominal muscles that would affect signal integrity [41]. However, high to excellent VAF_muscle_ was observed in the second day.

Individual EMG patterns were highly correlated across sets and days (sets: (0.89, 0.99); days: (0.89, 0.97), revealing similar patterns for all muscles, with some minor shifts that may be associated with the low proficiency of the subjects in the task. Namely, shifts in upper-limb muscles (TS, PM and BB) and lower-limb extensors (BF, ST and GL) were observed between sets, reflecting for unexperienced participants an inter-set variability in the activation of muscles during the pull, looking for better strategies to perform the lift.

The muscle synergy vectors and the synergy activation coefficients showed strong correlation between sets. Synergy activation coefficients had *rmax* values above 0.93 for intra-day analysis, suggesting that basic temporal activations are consistent. However, for synergy #2 in day one, a significant time shift was found in inter-set (−0.74 ± 4.35%) analysis. This may reveal that unexperienced participants with low refinement in the task may look for different adjustments in muscle activation when performing the power clean. We should note that the shift represents a small variation and the statistical differences between sets are due to one particular participant that presented a considerable shift. This statistical difference in time shift was not observed in day two, which suggests that the referred participant stabilized the movement technique in what concerns to the upper-limb synergy. Muscle synergy vectors had strong correlation and remained stable across sets. The vectors of synergy #3 had higher values of correlation in the second day, in line with the presented information regarding VAF_muscle_ intra-day reliability.

### 4.2. Inter-Day Reliability

The 5 RM test showed an excellent reliability with a near 1 ICC-value and a low SEM-value. These results are in line with previous studies that reported that multiple repetition maximum tests of traditional strength training exercises are reliable in recreational athletes [42,43].

The inter-day reliability of total VAF, using ICC (3,1), was moderate. When comparing both days, similar SEM-values to the inter-set values were observed (sets: (0.01, 0.02); days: (0.01, 0.03)). Thus, although ICC-values for inter-day reliability analysis are just moderate, the low SEM-values associated with absolute reliability represent a 0.01–0.03 of scatter in VAF-values around the actual score [37]. Regarding VAF_muscle_ ICC-values, almost all the muscles presented poor values, except TB and Gmax. The mean values of the sets for each day were similar and SEM values were low, ranging between 0.03 and 0.10, with exception of EDC, LD, RA and TA. RA presents the same limitations of OE, mainly regarding the adipose tissue of the abdominal region [41]. The other three muscles’ lack of reliability may be associated with EMG technique limitations in dynamic tasks, possibly due to crosstalk with neighbor muscles. LD EMG signal may be overestimated, reflecting crosstalk from the ES [44], EDC EMG signal may vary regarding the crosstalk from other forearm muscles [45], and TA may reflect crosstalk from gastrocnemius muscles [46]. However, for both days, almost every muscle accounted for > 75% of VAF_muscle_, with exception of RA and TA, that showed low-values of VAF_muscle_, meaning that the variability in the dataset is not accounted adequately by the two muscles. The lower reliability-values of VAF_muscle_ compared with total VAF are in line with the results of Kristiansen and colleagues [28] and Taborri, Palermo and colleagues [29]. Interestingly, when comparing individual EMG patterns between days, no shifts were found. This may be caused by the averaging of the paired sets correlations that eventually affect the higher and lower values of between-day sets comparison.

Synergy activation coefficients were strongly correlated and did not present any significant shift in time, i.e., subjects presented generally the same synergy activation timings across days. However, muscle synergy vectors just had moderate correlation between first and second session, which may reflect variations of the relative weighting of each muscle within each synergy. These findings are in line with the results presented by Kristiansen and colleagues [28] regarding the reliability of muscle synergies in bench press. The first obvious explanation may be related to EMG electrode placements across sessions, considering that slight deviations in position and orientation to the muscle fibers may cause modifications of the EMG signal. Moreover, as suggested by Kristiansen and colleagues [28], the lower correlation of muscle vectors may be due to the number of points utilized in the compared time series (equal to the number of muscles = 16) that are considerably less than the 200 time points utilized in the activation coefficients comparison. In addition, a learning effect may be present from the first to the second session, in which the redundancy of the musculoskeletal system and the optimization process minimizing motor effort cost may cause one altered recruitment option [2,47]. Considering that muscle synergies can be recruited by single neural commands, representing just one variable that may be controlled [6], the temporal activation of those commands will be consistent when performing a task [29], and consequently, the synergy activation coefficients reliability will be stronger than the reliability of muscle synergy vectors [28]. The moderate reliability of muscle vectors in a complex task may also be associated with the different levels of learning of the individuals and with the training background of each one.

**Figure 3:**
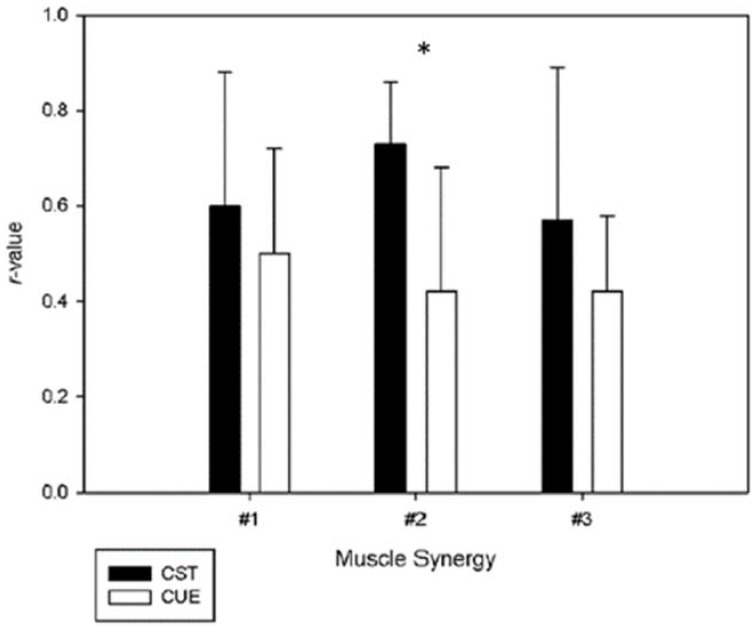
Muscle synergy vectors comparison between participants with (CST) and without (CUE) anterior background in general strength training. (*) represents significative differences between the two groups.

Although out of the scope of the present study, we performed a detailed analysis accounting for the prior experience in general strength training to further understand possible weaker correlations of muscle vectors between days. This resulted in two groups of six participants each. One group was represented by the participants with experience in general strength training, and the other comprised the participants with no experience at all. For the experienced group, the correlation-values of the vectors were 0.60 ± 0.29, 0.73 ± 0.13 and 0.57 ± 0.42, for synergy #1, #2 and #3, respectively. For the unexperienced group, the correlation-values were 0.50 ± 0.22, 0.42 ± 0.26 and 0.42 ± 0.16, for synergy #1, #2 and #3, respectively. This information is depicted in Figure 3. Overall, we observed that the muscle vectors of the experienced participants were more reliable than the muscle vectors of the group without any experience in strength training for the three-synergy model. Furthermore, the correlation of muscle vector #2 of the experienced group changed from moderate to strong. Thus, we expect that strength training may create adaptations that provide a greater capacity to adapt and to stabilize quickly in different demanding tasks for the neuromusculoskeletal system. This ‘transfer of learning’ may occur in a positive way when training one task, or in this case a number of various strength training traditional exercises, contribute to an increase in motor performance during a subsequent task, the power clean [48]. This suggests that previous developed coordination strategies shared with other acquired tasks may ensure that muscle synergies composition be flexibly exploited by individuals during skill acquisition [49]. Furthermore, the training strategies adopted to improve coordination in strength training exercises may influence the adaptations in muscle synergies. Previous work in bench press showed that pre-exhaustion methods and training programs with isolated exercises targeting weak muscle groups may change the patterns of muscle activity, namely in what concerns to the individual contribution of each muscle, and it should be relevant to understand the impact of these strategies in the neural adaptations to strength training overtime [50,51,52].

### 4.3. Limitations

As suggested by Kristiansen and colleagues [28], bilateral recording of EMG could support the study conclusions. Moreover, kinematic data could provide information regarding the muscle activation and the contribution of each muscle synergy during the movement, which could possibly relate the contribute of muscles along time and their impact in changes of kinematic variables, e.g., joint angles and velocities. It is important to understand that although there is well-documented evidence that muscle synergies reflect neural low-dimensional modules [6], some studies suggest that task constraints may influence the extracted muscle synergies by mathematic procedures [2,13]. When comparing with other studies using NMF caution should be taken because it may differ the synergy extraction methodology [53], VAF definition criteria [23], low-pass filtering [40] and chosen muscles [39].

## 5. Conclusions

This study showed that the individual and synergistic organization of muscles during the power clean remained the same between sets and days in participants with low level of expertise in the movement. Furthermore, synergy components were revealed to have strong correlation inter-set values and moderate (vectors) to strong (coefficients and individual EMG) inter-day correlation values. This information may reveal the robustness of the muscle synergy extraction procedure, establishing a relation between the mathematical output with the neurophysiological organization and adaptation of motor control. Thus, in the first instance, further investigation regarding strength and power training whole-body exercises should be conducted. This could provide information about how unexperienced individuals and weightlifters differ concerning coordination strategies, and how the highly trained individuals may develop individual neural strategies of motor control.

## Figures and Tables

**Table 1:** Intraclass correlation (ICC), SEM and Confidence Interval (CI (95%)) for of intra- (day 1 and 2) and inter-day reliability analysis of VAF and VAF_muscle_.

		VAF			VAF Muscle															
		#1	#2	#3	TS	PM	BB	TB	FDS	EDC	LD	ES	RA	TA	Gmax	VL	BF	ST	GL	OE
Intra-day 1	ICC (3,4)	0.95	0.94	0.92	0.92	0.91	0.82	0.89	0.91	0.97	0.94	0.80	0.89	0.84	0.97	0.84	0.94	0.89	0.92	0.65
	SEM	0.02	0.01	0.01	0.03	0.08	0.03	0.05	0.05	0.03	0.07	0.06	0.14	0.09	0.03	0.10	0.03	0.04	0.05	0.10
	CI (95%)	0.87 0.98	0.84 0.98	0.81 0.97	0.82 0.98	0.78 0.97	0.56 0.94	0.74 0.97	0.77 0.97	0.91 0.99	0.87 0.98	0.52 0.94	0.70 0.97	0.60 0.95	0.92 0.99	0.60 0.95	0.85 0.98	0.72 0.97	0.82 0.98	0.13 0.90
Intra-day 2	ICC (3,4)	0.98	0.97	0.92	0.98	0.97	0.94	0.97	0.95	0.97	0.95	0.94	0.92	0.93	0.95	0.88	0.88	0.81	0.95	0.89
	SEM	0.01	0.01	0.00	0.02	0.03	0.02	0.03	0.03	0.05	0.06	0.03	0.07	0.07	0.03	0.04	0.02	0.05	0.04	0.05
	CI (95%)	0.95 0.99	0.93 0.99	0.81 0.98	0.95 0.99	0.91 0.99	0.84 0.94	0.92 0.99	0.88 0.99	0.91 0.99	0.87 0.99	0.84 0.98	0.76 0.98	0.81 0.98	0.87 0.98	0.70 0.96	0.69 0.96	0.52 0.94	0.87 0.99	0.70 0.97
Inter-day	ICC (3,1)	0.66	0.62	0.54	0.09	0.43	0.48	0.83	0.24	0.13	0	0.09	0.42	0.30	0.63	0.29	0	0.26	0.38	0.19
	SEM	0.03	0.02	0.01	0.07	0.10	0.03	0.04	0.09	0.15	0.20	0.08	0.18	0.11	0.06	0.08	0.07	0.07	0.09	0.09
	CI (95%)	0.14 0.90	0.07 0.88	0.06 0.85	−0.52 0.63	−0.19 0.81	−0.13 0.83	0.49 0.95	−0.39 0.72	−0.51 0.68	−0.69 0.44	−0.51 0.64	−0.24 0.82	−0.34 0.75	0.08 0.88	−0.34 0.74	−0.72 0.38	−0.37 0.73	−0.25 0.79	−0.43 0.69

**Table 2:** Intra- and inter-day similarity values (*rmax*) and lag times (% of the power clean cycle) of synergy activation coefficients and individual EMG profiles. Bold values represent significantly differences from zero and Cohen’s d above 0.8.

	Intra-Day | Day 1				Intra-Day | Day 2				Inter-Day			
	% lag	*p*	*d*	*r_max_*	% lag	*p*	*d*	*r_max_*	% lag	*p*	*d*	*r_max_*
Individual EMG Profiles												
TS	−0.22 ± 0.46	**0.05**	−0.58	0.98 ± 0.01	−0.33 ± 0.47	**0.04**	−0.61	0.98 ± 0.01	−0.22 ± 0.64	0.30	−0.33	0.94 ± 0.02
PM	−0.19 ± 0.33	**0.03**	−0.63	0.96 ± 0.03	−0.21 ± 0.30	**0.04**	−0.61	0.98 ± 0.01	−0.21 ± 0.63	0.21	−0.38	0.90 ± 0.08
BB	−0.30 ± 0.45	0.05	−0.63	0.98 ± 0.00	−0.37 ± 0.40	**0.02**	**−0.88**	0.99 ± 0.01	0.09 ± 0.61	0.65	0.14	0.95 ± 0.04
TB	−0.18 ± 0.48	0.24	−0.36	0.98 ± 0.01	−0.22 ± 0.42	0.13	−0.50	0.98 ± 0.01	0.29 ± 0.76	0.26	0.36	0.95 ± 0.02
FDS	−0.18 ± 0.45	0.21	−0.39	0.98 ± 0.01	−0.33 ± 0.60	0.12	−0.52	0.98 ± 0.02	−0.59 ± 1.65	0.44	−0.23	0.93 ± 0.05
EDC	−0.19 ± 0.38	0.21	−0.40	0.98 ± 0.01	−0.07 ± 0.30	0.53	−0.20	0.97 ± 0.02	4.43 ± 9.05	0.05	0.58	0.92 ± 0.04
LD	−0.01 ± 0.02	0.32	−0.29	0.96 ± 0.02	−0.06 ± 0.15	0.18	−0.40	0.96 ± 0.02	1.86 ± 5.05	0.14	0.45	0.89 ± 0.08
ES	0.00 ± 0.00	1.00	0.00	0.98 ± 0.01	0.00 ± 0.00	1.00	0.00	0.98 ± 0.01	5.43 ± 17.16	0.32	0.30	0.96 ± 0.03
RA	−0.30 ± 0.53	0.13	−0.49	0.95 ± 0.02	−0.02 ± 0.23	0.84	−0.06	0.96 ± 0.02	7.30 ± 17.72	0.11	0.48	0.92 ± 0.04
TA	−0.15 ± 0.76	0.54	−0.18	0.97 ± 0.01	−0.20 ± 040	0.15	−0.47	0.96 ± 0.01	0.88 ± 1.74	0.14	0.48	0.93 ± 0.03
Gmax	−0.06 ± 0.35	0.56	−0.17	0.98 ± 0.01	−0.08 ± 0.29	0.67	−0.13	0.98 ± 0.01	0.05 ± 0.83	0.84	0.06	0.94 ± 0.05
VL	−0.03 ± 0.17	0.71	−0.11	0.99 ± 0.00	−0.07 ± 0.13	0.11	−0.48	0.99 ± 0.00	2.63 ± 8.67	0.89	−0.04	0.97 ± 0.01
BF	−6.87 ± 3.27	**<0.001**	**−2.01**	0.92 ± 0.03	−8.80 ± 3.48	**<0.001**	**−0.86**	0.93 ± 0.02	−0.12 ± 0.48	0.44	−0.24	0.96 ± 0.02
ST	−0.77 ± 0.94	**0.03**	−0.75	0.94 ± 0.02	−0.62 ± 0.71	**0.02**	**−0.83**	0.94 ± 0.03	1.87 ± 5.74	0.80	0.08	0.95 ± 0.04
GL	−6.45 ± 8.81	**0.03**	−0.61	0.89 ± 0.01	−5.16 ± 8.01	0.07	−0.61	0.89 ± 0.02	1.22 ± 2.80	0.20	0.39	0.93 ± 0.05
OE	−0.15 ± 0.43	0.29	−0.32	0.97 ± 0.01	−0.06 ± 0.21	0.42	−0.26	0.97 ± 0.01	4.82 ± 14.15	0.12	0.47	0.93 ± 0.04
Synergy Activation Coefficients												
#1	−0.30 ± 0.85	0.11	−0.46	0.97 ± 0.03	0.28 ± 1.45	0.71	−0.11	0.95 ± 0.05	0.07 ± 1.12	0.69	−0.12	0.87 ± 0.08
#2	0.74 ± 4.35	**0.05**	−0.58	0.97 ± 0.02	−0.37 ± 0.78	0.17	−0.45	0.97 ± 0.02	−0.92 ± 2.26	0.07	−0.54	0.90 ± 0.06
#3	−1.53 ± 4.76	0.37	−0.26	0.93 ± 0.05	−1.56 ± 3.26	0.08	−0.54	0.96 ± 0.04	−1.32 ± 16.92	0.14	−0.45	0.87 ± 0.08

**Table 3:** Intra- and inter-day similarity values (*r*) of muscle synergy vectors.

	Intra-Day		Inter-Day
	Day 1	Day 2	
Muscle Synergy Vectors			
#1	0.84 ± 0.19	0.83 ± 0.24	0.56 ± 0.27
#2	0.85 ± 0.20	0.87 ± 0.13	0.59 ± 0.25
#3	0.74 ± 0.24	0.86 ± 0.20	0.50 ± 0.27
